# Supplementary figures and images for: Osteology of Batrachuperus londongensis (Urodela, Hynobiidae): study of bony anatomy of a facultatively neotenic salamander from Mount Emei, Sichuan Province, China
Source: PeerJ. 2018 Mar 28;6:e4517. doi: 10.7717/peerj.4517 (PMC5878659; doi:10.7717/peerj.4517)

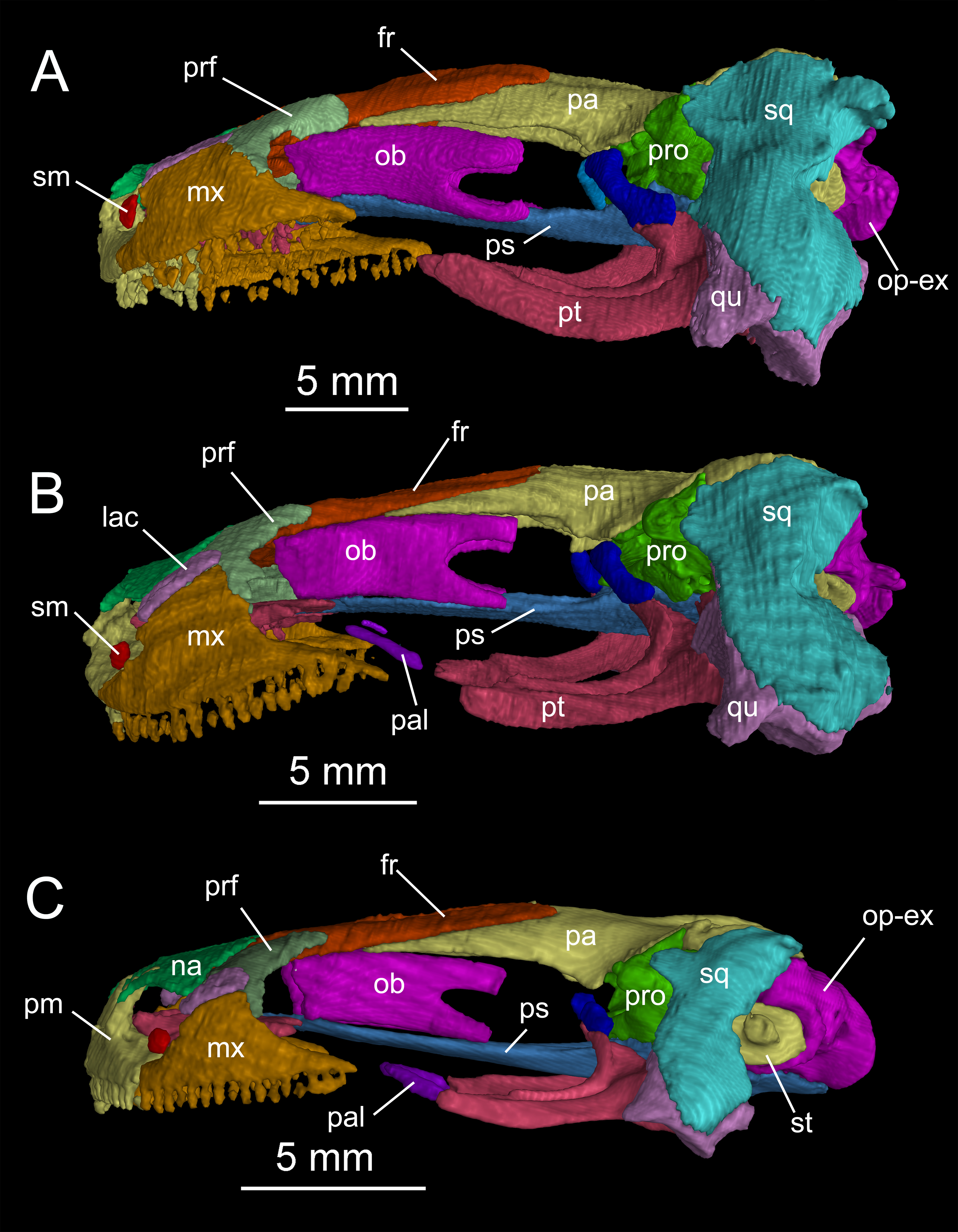

Supplement: Supplemental Information 1 — (A) CIB 65I0013/14380; (B) CIB 14381; (C) CIB 14482. Abbreviations used in this and other supplementary figures see Materials & Methods in main text. [file peerj-06-4517-s001.jpg]

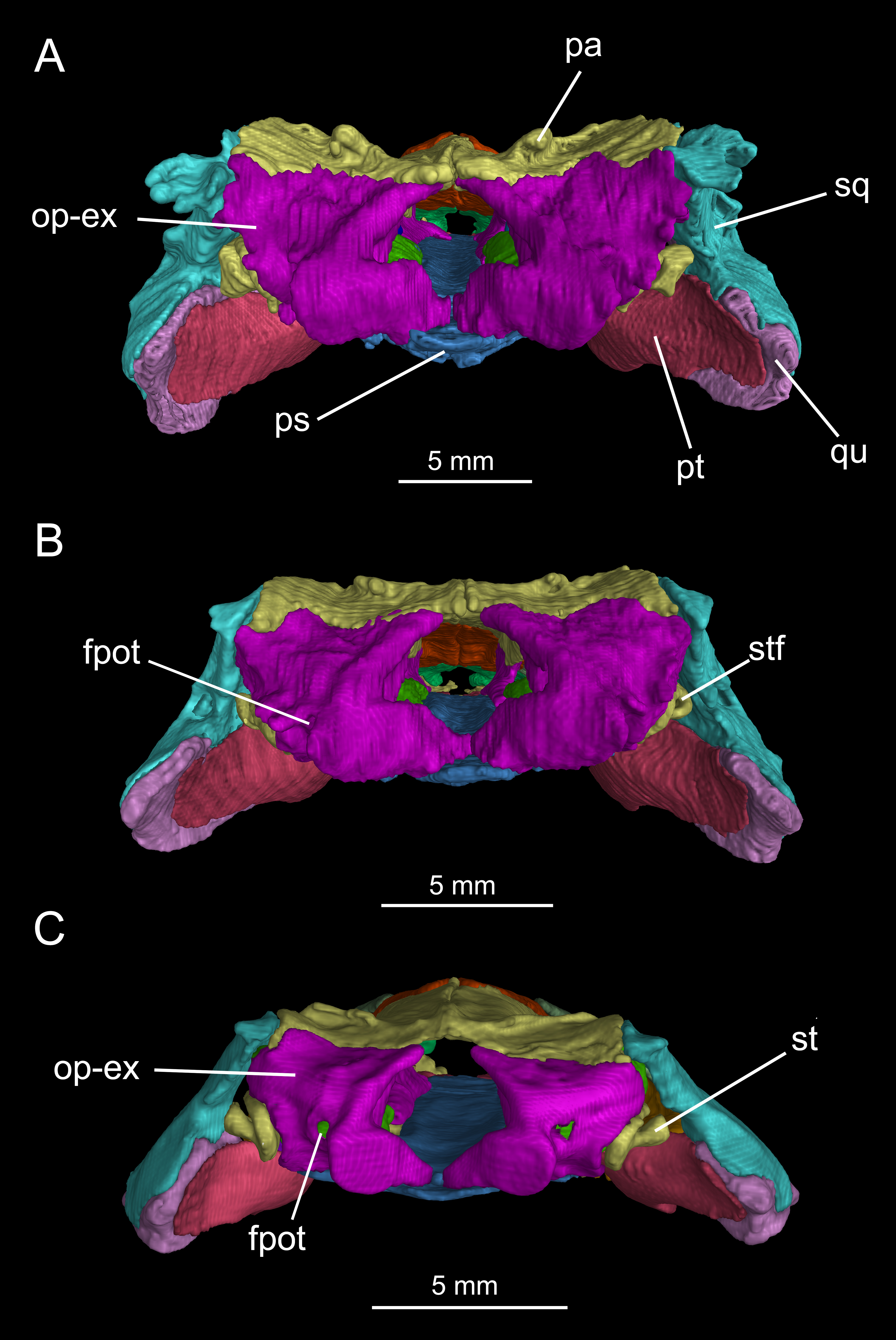

Supplement: Supplemental Information 2 — (A) CIB 65I0013/14380; (B) CIB 14381; (C) CIB 14482. [file peerj-06-4517-s002.jpg]

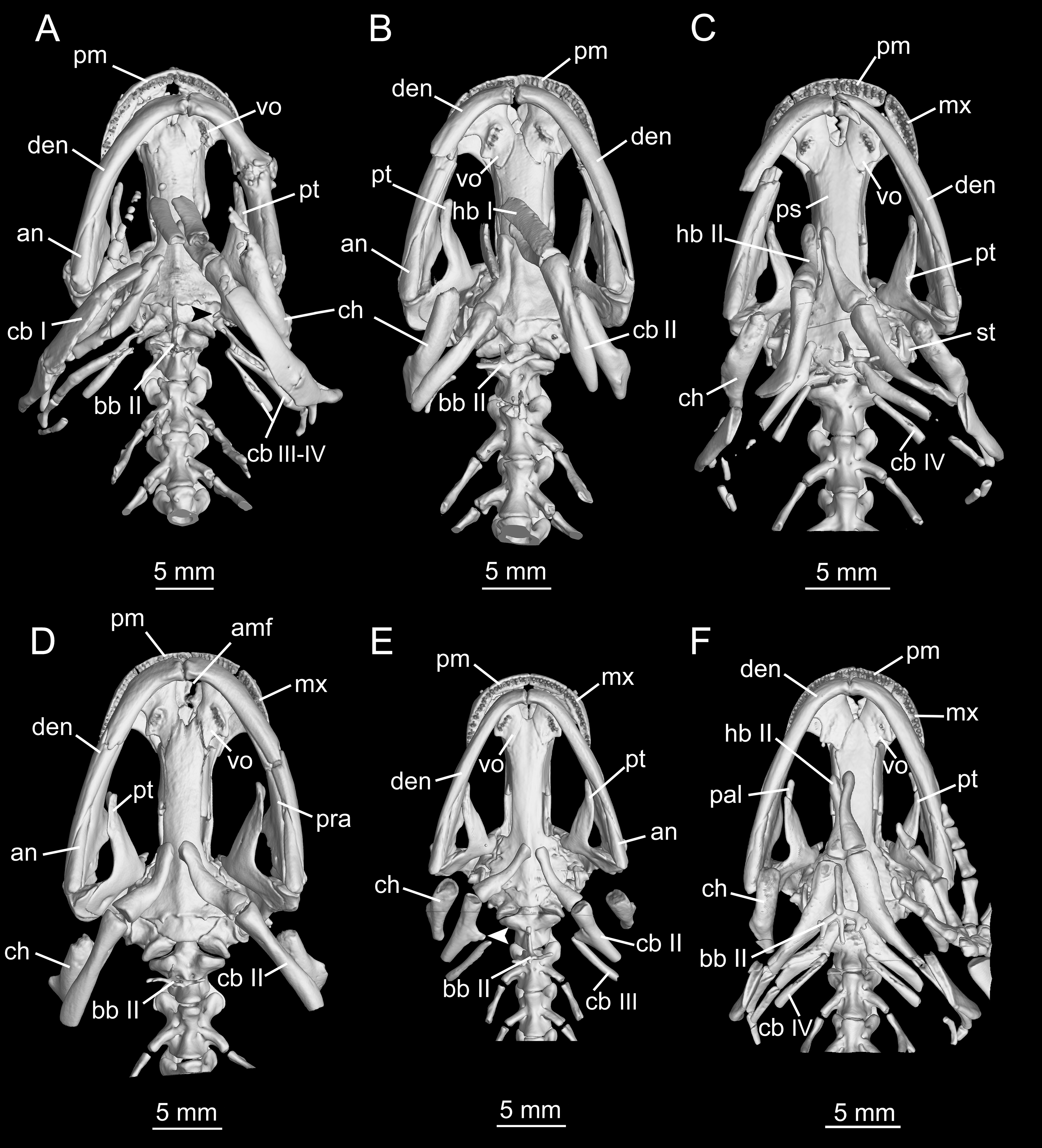

Supplement: Supplemental Information 3 — (A) CIB 65I0013/14380; (B) CIB 14504; (C) CIB 14484; (D) CIB 14507; (E) CIB 14487; (F) CIB 14482. Arrow in (A) and (E) pointing to the prominent process projecting from the midlength of the ceratobranchial II for attachment of the subarcualis rectus II muscle. [file peerj-06-4517-s003.jpg]

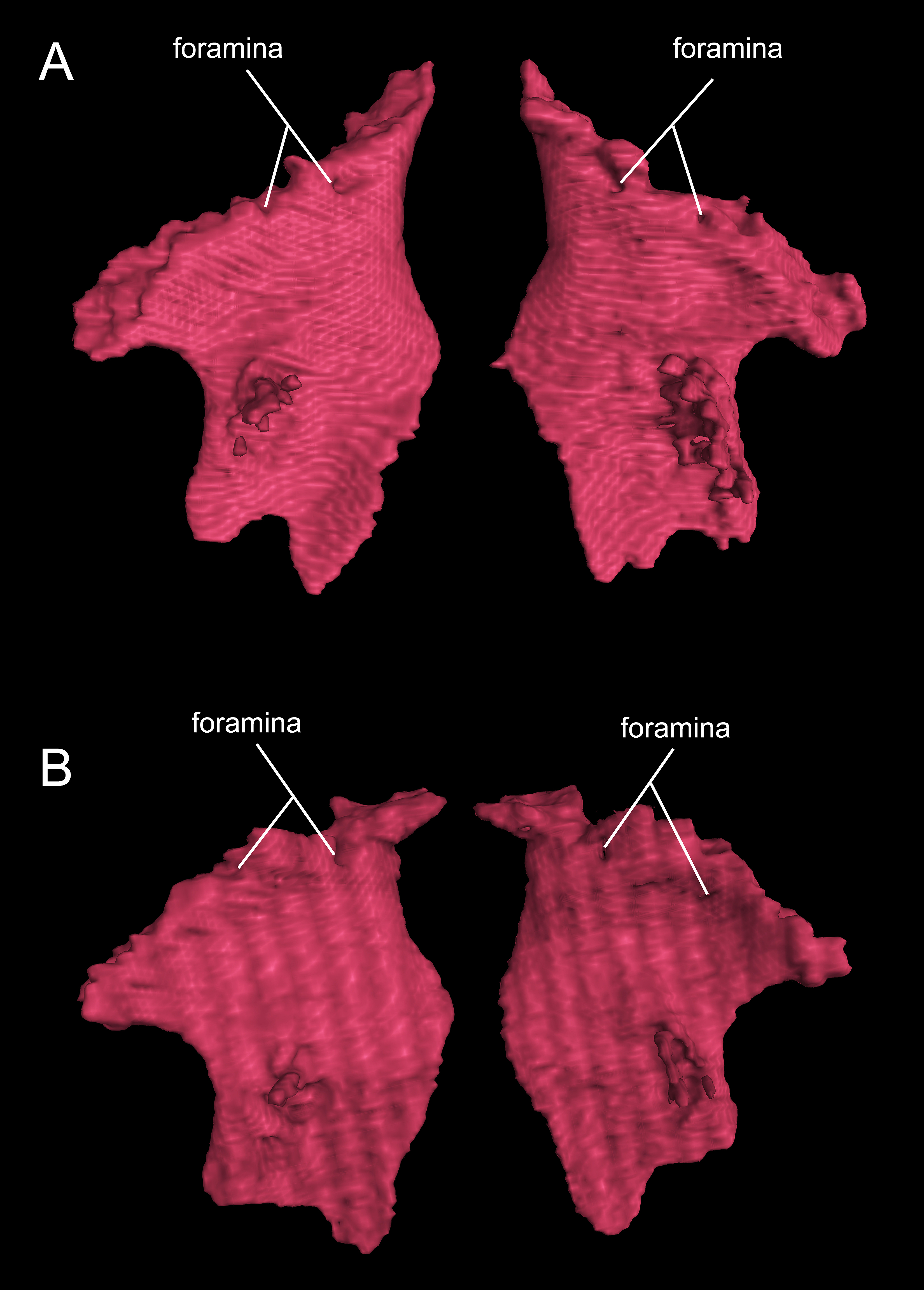

Supplement: Supplemental Information 4 — (A) CIB 65I0013/14380; (B) CIB 14381. Foramina labeled in images are for passage of the ramus ventralis of the trigeminal nerve (CN V) and the ramus palatinus of the facial nerve (CN VII) as described in main text. [file peerj-06-4517-s004.jpg]

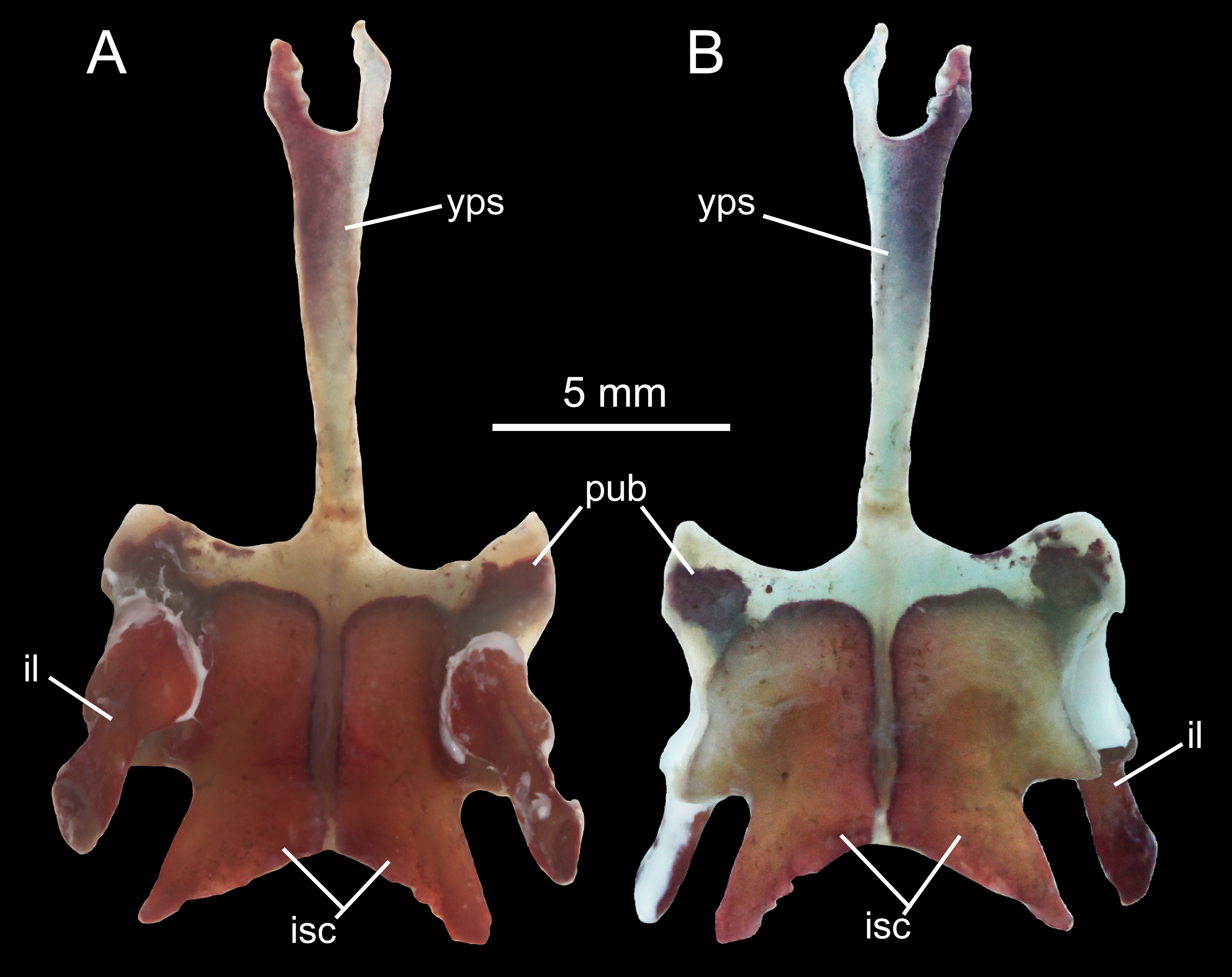

Supplement: Supplemental Information 5 [file peerj-06-4517-s005.jpg]
