# Supplementary material for: Osteology of Batrachuperus londongensis (Urodela, Hynobiidae): study of bony anatomy of a facultatively neotenic salamander from Mount Emei, Sichuan Province, China
Source: PeerJ. 2018 Mar 28;6:e4517. doi: 10.7717/peerj.4517 (PMC5878659; doi:10.7717/peerj.4517)
Supplement: Supplemental Information 6 [file peerj-06-4517-s006.docx]

Table S1. Specimens used for comparison in this study

| Taxon Name | Catalogue Number | Date of Collection | Type Status | Locality |
| --- | --- | --- | --- | --- |
| *Batrachuperus karlschmidti* | FMNH 49380 | August 10, 1943 | Paratype | Chiala, Luhohsien, Sichuan Province, China |
| *Batrachuperus pinchonii* | FMNH 170703 | August 9, 1939 | Paratype | Lianghokou, Muping, Sichuan Province, China |
| *Batrachuperus taibaiensis* | CIB 20040235 | February 2004 | Referred specimen | Laoxiancheng, Zhouzhi, Shaanxi Province, China |
| *Batrachuperus tibetanus* | FMNH 5901 | December 1923 | Paratype | Tibetan border of Gansu Province, China |
| *Batrachuperus yenyuanensis* | FMNH 49371 | July 4, 1942 | Paratype | Bailingshan, Sichuan Province, China |
| *Paradactylodon mustersi* | FMNH 211936 | March 9, 1978 | Referred specimen | Pa-in tributary of Paghman stream, Kabul, Afghanistan |
| *Pseudohynobius flavomaculatus* | CIB 17344 | June 15, 1979 | Referred specimen | Hanchi, Lichuan, Hubei Province, China |
